# Rapid HIV-1 drug resistance testing in a resource limited setting: the Pan Degenerate Amplification and Adaptation assay (PANDAA)

**DOI:** 10.11604/pamj.2021.40.57.28558

**Published:** 2021-09-22

**Authors:** Vinie Kouamou, Chiratidzo Ellen Ndhlovu, David Katzenstein, Justen Manasa

**Affiliations:** 1 Unit of Internal Medicine, Faculty of Medicine and Health Sciences, University of Zimbabwe, Harare, Zimbabwe,; 2 Department of Molecular Virology, Biomedical Research and Training Institute, Harare, Zimbabwe,; 3 Department of Medical Microbiology, Faculty of Medicine and Health Sciences, University of Zimbabwe, Harare, Zimbabwe

**Keywords:** HIV-1, ART, pre-treatment drug resistance, HIV drug resistance, genotyping assays, resource limited settings

## Abstract

**Introduction:**

pre-treatment drug resistance (PDR) can compromise the 3^rd^ 95-95-95 global target for viral load suppression. The high complexity and cost of genotyping assays limits routine testing in many resource limited settings (RLS). We assessed the performance of a rapid HIV-1 drug resistance assay, the Pan Degenerate Amplification and Adaptation (PANDAA) assay when screening for significant HIV-1 drug resistance mutations (DRMs) such as K65R, K103NS, M184VI, Y181C and G190A. **Methods:** we used previously generated amplicons from a cross-sectional study conducted between October 2018 and February 2020 of HIV-1 infected antiretroviral therapy (ART)-naïve or those reinitiating 1^st^ line ART (18 years or older). The performance of the PANDAA assay in screening K65R, K103NS, M184VI, Y181C, and G190A mutations compared to the reference assay, Sanger sequencing was evaluated by Cohen´s kappa coefficient on Stata version 14 (StataCorp LP, College Station, TX, USA).

**Results:**

one hundred and twenty samples previously characterized by Sanger sequencing were assessed using PANDAA. PDR was found in 14% (17/120). PDR to non-nucleoside reverse transcriptase inhibitors (NNRTIs) was higher at 13% (16/120) than PDR to nucleotide reverse transcriptase inhibitors (NRTIs), 3% (3/120). The PANDAA assay showed a strong agreement with the reference assay, i.e. Sanger sequencing for all five target DRMs (kappa (95%CI); 0.93 (0.78-0.98)) and NNRTI DRMs (kappa (95%CI); 0.93 (0.77-0.980), and a perfect agreement for NRTI DRMs (kappa (95%CI); 1.00 (0.54-1.00)).

**Conclusion:**

the PANDAA assay is a simple and rapid method to identify significant HIV DRMs in plasma samples as an alternative to Sanger sequencing in many RLS.

## Introduction

Human immunodeficiency virus (HIV) drug resistance (HIVDR) is a serious threat to the global scale-up of HIV treatment. In resource limited settings (RLS), limited access to viral load (VL) monitoring and genotypic resistance testing make managing HIV more difficult. These factors contribute to virologic failure and development of drug resistance mutations (DRMs) [1-3]. High rates of acquired and pre-treatment drug resistance (PDR) to non-nucleoside reverse transcriptase inhibitor (NNRTI) have previously been reported in Zimbabwe [4-6]. Most people living with HIV (PLHIV) in Zimbabwe have been on a standard NNRTI-based 1^st^ line antiretroviral therapy (ART), either efavirenz (EFV) or nevirapine (NVP) in combination with tenofovir disoproxil fumarate (TDF) and lamivudine (3TC) at some time during the course of their treatment. However, in May 2019, Zimbabwe introduced dolutegravir (DTG) in 1^st^ line ART regimens in response to the recent WHO guidelines for countries whose national PDR had reached > 10% [6]. Dolutegravir in combination with TDF and 3TC has been given as a fixed dose combination (TLD) to ART naïve individuals initiating treatment and to virologically suppressed ART experienced people.

Genotypic resistance testing by the gold standard Sanger sequencing is not widely available in Zimbabwe because of high test costs, limited laboratory capacity and high capital investment required to set up the laboratories. However, the amplification of the HIV pol gene by polymerase chain reaction (PCR) has been accomplished in several laboratories in the country [4, 7-10]. Commercial laboratories in South Africa, United States of America and United Kingdom offer diagnostic sequencing from plasma, but this is expensive and the turn-around-time for results is 1-2 weeks. Therefore, in most instances, clinically based ART switches are practised. Thus, such switching may occur unnecessarily or individuals may be switched to suboptimal treatment leading to the accumulation of resistance mutations [5, 11, 12].

The World Health Organization (WHO) has prioritized expanding laboratory capacity in many RLS to improve access to HIVDR testing. Several groups have developed point mutation assays (PMAs) [13-17] that detect key DRMs (K65R and M184V for NRTIs; and K103NS, V106AM, Y181C, and G190A for NNRTIs) which are found in 98.8% of patients failing NNRTI-based 1^st^ line regimens [18, 19]. Similarly, considerable work has previously been done in the development of low-cost reagents for Sanger sequencing for RLS [20, 21]. The Pan Degenerate Amplification and Adaptation (PANDAA) assay has previously been described [14]. Briefly, the PANDAA assay is an allelic discrimination test designed with differentially labeled TaqMan probes to discriminate wild-type DNA (K65, M184, K103, Y181 and G190) from the DRMs (substitution at a specific codon position by the mutant amino acid known as K65R, M184VI, K103NS, Y181C and G190A). The PANDAA assay has recently been successful in detection of these acquired DRMs among adolescents and young adults failing ART in Zimbabwe [5]. This current study sought to assess the performance of PANDAA in screening for PDR among adults initiating or re-initiating NNRTI-based 1^st^ line ART.

## Methods

### Study design, population and setting

We used previously generated amplicons from a cross-sectional study conducted between October 2018 and February 2020. This was a study of HIV-1 infected consenting participants (18 years or older) who presented to the Parirenyatwa Hospital HIV ART treatment clinic in Harare, Zimbabwe. Consenting participants were ART-naïve or reinitiating NNRTI-based 1^st^ line ART after reporting previous exposure to ART (prior ART exposed), but having defaulted ART for at least 3 months. The amplicons were batched and stored at -20°C for 7 months prior to being assayed with PANDAA. The performance of the PANDAA assay in detecting DRMs with standard genotyping resistance testing by Sanger sequencing as the reference method was assessed.

### Laboratory testing

The PANDAA assay differentiates the wild type allele 2 (labeled VIC) and the individual allele 1 (labeled FAM) coding for each DRM (K65R, K103NS, M184VI, Y181C, and G190A). The CFX96TM Real-Time system (Bio-Rad Laboratories, Inc., CA, USA) was used to test for all 5 codons in every sample. For the PANDAA assay, the amplicons were added to the qPCR master mix containing probes (VIC-labeled wild-type and FAM-labeled DRM-specific probes) and forward and reverse PANDAA primers and performed as previously described by MacLeod *et al*. (2019) [14]. Each run was performed with a control, which served as quality assessment for the PANDAA assay. The controls used in this study included control 1a (50%/50%) containing 50% DRM and 50% wild-type at 1.0 x 10^5^ copies/L and control 1b (100%) containing 0% DRM or a wild type at 1.0 x 10^5^ copies/L. Data generated by the CFX96TM Real-Time system for each sample were exported to Microsoft Excel for analysis. The relative abundance of the wild type versus the mutant codon was calculated based on the CT values of each fluorophore at the appropriate wavelength. The PANDAA assay as performed at the University of Zimbabwe, required approximately 1 hour 45 mins from the amplicons input to the analyzed result.

### Statistics and data analysis

The results for each sample were classified by the detection of DRMs as either wild-type or mutant at codon K65R, Y181C, M184VI, K103NS and G190A. DRMs detected by PANDAA and not by Sanger were defined as false-positive and those with DRMs confirmed by Sanger sequencing but not detected by the PANDAA assay were defined as false-negative. The Cohen´s kappa coefficient, implemented in Stata version 14 (StataCorp LP, College Station, TX, USA) determined the level of agreement between Sanger genotyping and PANDAA. The kappa coefficient was interpreted as: 0.41 to 0.60, moderate agreement; 0.61 to 0.80, substantial agreement; 0.81 to 1.00, strong or almost perfect agreement. Additional DRMs only detected by Sanger to NRTIs (L74I, D67N, K70E and K219R) and NNRTIs (V106M, K101E and P225H) were described using the Stanford HIV database [22]. Socio-demographic characteristics (age and gender) and clinical data (CD4+ cell count and VL) were extracted from the medical record.

### Ethical considerations

The study was reviewed and approved by the local institutional review board of the Joint Research and Ethics Committee of the University of Zimbabwe and Parirenyatwa Group of Hospitals (JREC/250/18) and by the Medical Research Council of Zimbabwe (MRCZ/A/2418).

**Funding**: the author(s) received no financial support for the research, authorship, and/or publication of this article. This research was made possible through various local collaborations including the Department of Infectious Diseases Research Laboratory at the University of Zimbabwe and the Biomedical Research and Training Institute.

## Results

### Baseline demographic and clinical characteristics

Over half of the participants were female (55%). The median (IQR) age of the participants was 36 (30-46) years whilst the median (IQR) CD4 cell count and log10 VL were 207 (92-381) cells/µL and 4.91 (4.38-5.37) copies/mL respectively.

### Pre-treatment drug resistance (PDR)

Altogether, 120 amplicons genotyped by Sanger sequencing and stored at -200°C for 7 months were all successfully tested by PANDAA. Pre-treatment drug resistance (K65R, M184V, K103N, Y181C and G190A) among the 120 participants was found in 14% (17/120). PDR to NNRTI was higher, found in 13% (16/120) than PDR to NRTI, 3% (3/120). Besides DRMs assessed by PANDAA (K65R, M184V, K103N, Y181C and G190A), additional major DRMs to NRTI (L74I, D67N, K70E and K219R) were found in 3/120 participants (3%), as follows: L74I in combination with M184V were found in 2/120 participants (2%) and D67N +K70E+219R together with M184V occurred in 1 participant (1%). The mutation L74I is selected primarily by didanosine and abacavir (ABC) and occasionally by TDF; K219N/R are accessory thymidine analog mutations (TAMs) that usually occur in combination with multiple other TAMs; D67N is a non-polymorphic TAM associated with low-level resistance to zidovudine and stavudine and K70E cause low-level resistance to TDF and ABC. Similarly, additional major DRMs to NNRTIs (V106M, K101E and P225H) were present in 5/120 participants (4%), as follows: V106M (2 participants) is a non-polymorphic mutation particularly common in subtype C viruses that causes high-level resistance to NVP and EFV and low/intermediate resistance to doravirine; K101E (found in 1 participant) is a non-polymorphic primarily accessory mutation that causes intermediate resistance to NVP and low-level resistance to EFV and finally P225H (found in 2 participants) is a non-polymorphic EFV-selected mutation [22].

### Agreement between PANDAA and Sanger sequencing

The PANDAA assay showed a strong agreement with Sanger sequencing for all five target DRMs (kappa (95%CI); 0.93 (0.78-0.98)), NNRTI DRMs (kappa (95%CI); 0.93 (0.77-0.98)) and a perfect agreement for NRTI DRMs (kappa (95%CI), 1.00 (0.54-1.00)) (Table 1).

**Table 1:** performance of the PANDAA assay with Sanger sequencing as the reference method

Mutations	True positive	True negative	False positive	False negative	Kappa value (95% CI)
**Overall DRMs**	17	101	2	0	0.93(0.78-0.98)
					
**NRTI DRMS**	3	117	0	0	1.00(0.54-1.00)
K65R	0	120	0	0	#
M184V	3	117	0	0	1.00 (0.54-1.00)
**NNRTI DRMS**	16	102	2	0	0.93(0.77-0.98)
K103N	15	103	2	0	0.93(0.76-0.98)
Y181C	0	119	1	0	#
G190A	3	117	0	0	1.00(0.54-1.00)

# = not computed; CI= confidence interval; DRMs= drug resistance mutations; NRTI DRMs= frequency of nucleotide reverse transcriptase inhibitor mutations detected either individually or together (K65R and/or M184V); NNRTI DRMs= frequency of non-nucleoside reverse transcriptase inhibitor mutations detected either individually or together (K103N and/or Y181C and/or G190A).

## Discussion

HIV drug resistance (HIVDR) testing can assist in the selection of optimal ART regimens to attain the third 95-95-95 global target for VL suppression by 2030. However, limited laboratory capacity and high costs limit routine drug resistance testing in many RLS including Zimbabwe. To address the growing problems of HIVDR and following the most recent (2020) WHO HIV resistance network recommendations [23], several point mutations assays (PMAs) have been developed and evaluated for detection of HIVDR against NNRTIs-based 1^st^ line ART regimens. Here, we focused on assessing the performance of an HIVDR assay, the PANDAA assay, in detecting major PDR among adults initiating or re-initiating 1^st^ line ART in Zimbabwe. In this study, the PANDAA assay showed a strong agreement (k = 0.93) in detecting major PDR compared to the gold standard, Sanger sequencing. Similarly, we recently reported a high sensitivity and specificity (98% and 94% respectively) and a strong agreement of the PANDAA assay compared to Sanger sequencing in detecting acquired DRMs [5]. The findings in this study strengthen the case for the implementation and use of PANDAA assay as an alternative method to rapidly detect drug resistance in many RLS including Zimbabwe.

Point mutation assays are potentially simpler, faster, and lower-cost alternatives to sequencing in RLS. The Oligo-nucleotide Ligation Assay (OLA), to detect DRMs has recently (2020) demonstrated its ability to detect PDR to NNRTI-based ART in Kenya [24] and was previously implemented successfully in Thailand, Kenya and Zimbabwe [25-27]. Furthermore, point mutation assays require limited equipment and can detect minority-variant DRMs (< 20% of the viral population) often missed by Sanger sequencing [28]. In this study, the PANDAA assay required a quantitative real-time PCR technology (CFX96TM Real-Time System), that is accessible to molecular laboratories in Zimbabwe including the Newlands Clinic, the Infectious Diseases Research Laboratory and the Biomedical Research Training Institute. Unlike Sanger sequencing, bioinformatics analysis and specialized software are not required for PANDAA, the assay and analysis software are user-friendly. Importantly, the PANDAA testing of amplicons was conducted locally (at the University of Zimbabwe) in approximately 1h 45 mins, eliminating the need for shipping amplicons outside the country for genotyping. Similarly, the recent OLA-Simple, a lateral flow detection was designed to be manually readable [15] with in-house software which provided guidance for non-trained users. Panpradist *et al*. (2019) reported that the OLA-Simple equipment, reagent and personnel costs were less than other existing HIVDR assays.

Although many PLHIV in Zimbabwe are still on a NNRTI-based 1^st^ line regimens, Zimbabwe and many other low and middle-income countries (LMICs) has introduced the single tablet tenofovir disoproxil fumarate/lamivudine/dolutegravir (TLD) in 1^st^, 2^nd^ and 3^rd^ line ART. The increased distribution of lower cost TLD may minimize the need for pre-treatment and acquired NNRTI testing for HIVDR in LMICs as DTG has proven to have a high barrier to resistance and hence rarely selects for HIVDR in clinical trials [29, 30]. However, surveillance for drug resistance remains critical as the findings recently reported from the ADVANCE study provide an important note of caution. As reported by Siedner *et al*. (2020), among South African adults, NNRTI resistance prior to treatment was associated with long-term failure of integrase inhibitor-containing 1^st^ line regimens [31]. Hence, there may be need for screening for PDR to NNRTI among DTG initiators using rapid and easy PMAs such as PANDAA in many LMICs. Moreover, PANDAA and other PMAs are important for detecting NRTI DRMs as discussed in a recent systematic review [32] of the genetic mechanisms of dolutegravir resistance. In this review, Rhee *et al*. (2019) identified risk of functional monotherapy with implications for the use of DTG + 2 NRTIs in NRTI-experienced people in LMICs. In settings with limited access to VL testing and genotyping, optimized background therapy in PLHIV with virologic failure are limited [33]. Similarly, HIVDR mutations may be selected in people taking DTG monotherapy [34-37], suggesting that a fully active NRTI backbone may be needed to sustain effectiveness of 1^st^ line DTG-based regimens.

In RLS, implementation and monitoring of integrase strand transfer inhibitor-based regimens as more effective treatment for HIV may be limited by access to VL and genotypic resistance testing, which require stable power supply and real time PCR equipment. While the PANDAA may serve as simpler, alternative to detect DRMs, the diagnostic accuracy, (sensitivity and specificity) of the assay was not assessed due to the low prevalence of individual PDR. Therefore, larger sample sizes from population based surveys are required to cement our findings that, PANDAA could be used as a simple and rapid alternative approach to HIVDR assay in LMICs.

## Conclusion

The PANDAA assay as previously demonstrated addresses challenges in implementing HIVDR testing in LMICs. Thus, it could represent a simple and rapid alternative approach to HIVDR assay in LMICs.

### What is known about this topic

- The 2019 WHO HIV Resistance Network (HIVRESNET) annual meeting advocated for the implementation of point mutation assays for HIV drug resistance testing in resource limited settings;
- Several point mutation assays had been developed to detect HIV drug resistance, including the oligonucleotide ligation assay, the allele-specific primer extension, multiplexed melt curve analysis and the PANDAA assay;
- These point mutation assays can be used as an alternative to Sanger sequencing with a real-time thermal cycler in many resource limited settings.

### What this study adds

- Comparative HIV drug resistance detection between the PANDAA assay and Sanger sequencing demonstrated highly concordant detection of mutations;
- The PANDAA assay can be used as a rapid HIV-1 drug resistance testing in a resource limited setting for screening HIV-1infected persons initiating or re-initiating first-line antiretroviral therapy in a resource limited setting;
- Unlike Sanger sequencing, the PANDAA assay requires minimal laboratory equipment and no bioinformatics analysis is needed for resistance mutations results.
